# Underlying Principles of Natural Selection in Network Evolution: Systems Biology Approach

**Published:** 2007-09-26

**Authors:** Bor-Sen Chen, Wei-Sheng Wu

**Affiliations:** Lab of Control and Systems Biology, National Tsing Hua University, Hsinchu, 300, Taiwan

## Abstract

Systems biology is a rapidly expanding field that integrates diverse areas of science such as physics, engineering, computer science, mathematics, and biology toward the goal of elucidating the underlying principles of hierarchical metabolic and regulatory systems in the cell, and ultimately leading to predictive understanding of cellular response to perturbations. Because post-genomics research is taking place throughout the tree of life, comparative approaches offer a way for combining data from many organisms to shed light on the evolution and function of biological networks from the gene to the organismal level. Therefore, systems biology can build on decades of theoretical work in evolutionary biology, and at the same time evolutionary biology can use the systems biology approach to go in new uncharted directions. In this study, we present a review of how the post-genomics era is adopting comparative approaches and dynamic system methods to understand the underlying design principles of network evolution and to shape the nascent field of evolutionary systems biology. Finally, the application of evolutionary systems biology to robust biological network designs is also discussed from the synthetic biology perspective.

## Introduction

The notion of system-level understanding in biology has a long history (von Bertalanffy, 1993; Wiener, 1948), but has not received a large amount of genome sequencings until now. Genome sequences can tell us the whole static information of gene, regulatory regions, proteins, tRNAs, repeats and so on underlying DNA sequences (Lander, 1999). At the same time, the development of other high throughput technologies also provides us a comprehensive set of dynamic data: DNA microarrays, protein microarrays, ChIP-chip data and so forth. All these developments have transformed biological research from a data-poor state to a data-rich state and provided us an excellent opportunity to analyze biology on the system level (Schwikowski et al. 2000). Therefore, system theory and cell biology have enjoyed a long relationship, which in the context of systems biology have received renewed interest in recent years. Systems biology is concerned with the dynamic behavior of biochemical networks within cells and in cell populations. A principal goal of systems biology is to turn these static maps into dynamic models (Kitano, 2002).

For a long time, biologists have found that many biological functions and diseases cannot be explained by the function of an individual gene or protein. Instead, they should be the exhibition of an interactive network of protein-protein or protein with other molecules (Hartwell et al. 1999; Eisenberg et al. 2000). They also found that some particular characteristics of biological systems could display perfect adaptation and homeostatic regulation despite large changes in the environment or alternations in the internal parameters of the system (Alon et al. 1999; von Dassow et al. 2000). Such robustness undoubtedly is the result of the organism’s evolution over a long time to be able to adapt to the environmental changes. To understand the magic biological functions and robustness of biological networks, we have to integrate the information of genome sequences, mRNA expression, proteome and so on from the system-level point of view and to analyze the composition of biological systems on various levels: interactions among modules, system dynamics, underlying control methods and design principles in the evolutionary process (Tong, 2004).

**Figure 1.**
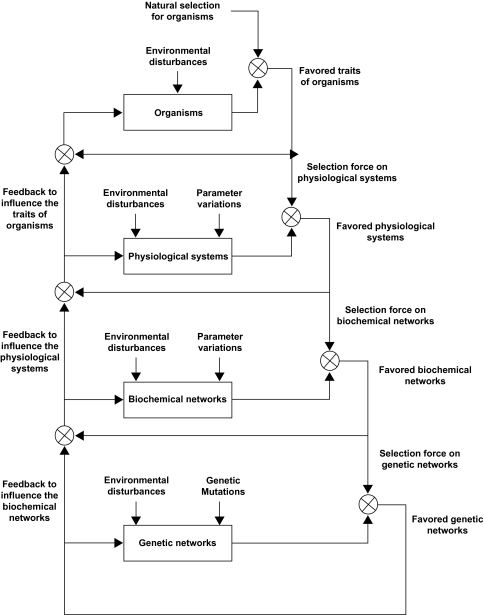
The natural selection process on the interplaying of hierarchical biological networks. The high-level selection will become the selection force on low level. The natural selection on organisms selects its favored organisms. Once the favored organisms are selected, the low-level biological networks have to maintain the favored physiological systems of the selected organisms. Hence, these favored organisms become the selection force to select their favored physiological systems. The favored physiological systems will lead to the selection force on biochemical networks. The favored biochemical networks by natural selection will become the selection force on genetic networks. On the other hand, the lower-level selected networks will feedback to influence the higher-level networks in evolution. Therefore, the natural selection actually acts on the interplaying of the multiple bionetworks.

The complexity of biological systems does not reside solely in the number of components and interactions, or in their associated structural and physicochemical properties, but in the hierarchical network connections across space and time scales from gene level to cell level to tissue level to organism level and finally to population level (see Fig. 1). Further, all biological systems are complex adaptive systems, in which high level networks reflect the collective dynamics of lower level networks and then feedback to affect high-level network dynamics (Medina, 2005). Evolutionary changes operate on multiple levels and multiple scales: from genetic networks, to biochemical networks, to physiological systems, to organisms, to populations, to communities and to the biosphere (Freeman and Herron, 2001; Medina, 2005; Koonin and Wolf, 2006). Although the lower-level networks govern the behavior of higher-level networks, the understanding of these lower-level networks is not sufficient for comprehending how macroscopic features emerge, how natural selection operates to lead to those features, where those features refer to the properties of the organism. How natural selection operates to modify the details, such as the rules that govern organismal development due to feedbacks from fitness differences among organisms? What features convey robustness to biological systems? How different should we expect the robustness of different biological systems to be, depending on whether natural selection is operating primarily on the whole system or on its parts? How does robustness trade off against adaptability? How does natural selection deal with environmental disturbances or noises? When does synchrony emerge and what is its implication for robustness? When and how does cooperative behavior emerge? These are the fundamental questions of biology from the evolution perspective (Stevens, 2004; Levin, 2006). This review tries to highlight these problems and to give some answers from the systems biology perspective.

Systems biology is not merely a contemporary manifestation of traditional biological engineering. The crucial difference is that the kinds of measurement and manipulation in modern systems biology is at the molecular level, and the data sets being generated and considered are highly multivariate because of the existence of high-throughput experimental assays at the genomic, proteomic, and metabolic levels. Systems biology aims at finding the true molecular and cellular mechanisms underlying the operation of biological systems, rather than phenomenological descriptions to which higher levels of organization (e.g. tissue, organ, and organism) are restricted (Wolkenhauer, 2001; Hood, 2003). Therefore, the approach of systems biology evolution is different from the conventional approach of biological evolution. In a study of conventional biological evolution, most contemporary phylogenetic trees are constructed from sequence data and, are usually developed from point mutations (substitutions, additions, or deletions) in a single gene. Each tree is an evolutionary hypothesis (Koonin and Wolf, 2006). Typically, to improve the confidence in the validity of any given tree, we use larger sets of sequence data from more organisms. Further, some notable microbiologists argue that phylogenetic trees represent inadequately the evolutionary history and that such trees do not do justice in portraying adequately the importance of the phenomena such as hybridization, endosymbiosis, recombination and horizontal gene transfer because these static trees cannot describe the underlying mechanisms of adaptive evolution (Kirschner, 2005).

At present, there are two prevailing interpretations of what systems biology is: (1) the integration of data obtained from experiments at various levels and associated with the “omics” of technologies and (2) the dynamic interactions of gene products, proteins and cells that bring about the structures and functions of cells, higher levels of the organization, such as tissues, organs and so on (Ideker et al. 2001; Ideker, 2004). The first view is more an informatics perspective, developing tools for data integration and fusion, while the second approach is motivated by data-based mathematical modeling and simulation. The first camp would often motivate their work by referring to a blood of data, while those interested in dynamic modeling of networks are concerned about the each of the quantitative data set. Therefore, the systems biology approach to biological network evolution is also discussed from two perspectives, i.e. data integration-based systems biological evolution and dynamic interaction-based systems biological evolution. They will be reviewed in the following paragraphs.

Because post-genomics research is taking place throughout the tree of life, comparative approaches offer a way for combining data from many organisms to shed light on the evolution and functions of biological systems from the gene to organismal levels (Graur and Li, 2000; Doolittle, 2005; Gelfand, 2006). Although comparative genomics has benefited from a long tradition of theoretical work by molecular evolutionists (Stearn and Magwene, 2003; Evangelisti and Wanger, 2004; Doolittle, 2005), new datasets provided by systems biology are offering theoreticians new ways to study evolutionary processes (Koonin and Wolf, 2006). Comparative studies can give insight into even the highest-level principles of life. For example, revolutionary findings in network theory have in part come from genomic data from a wide range of organisms, leading researchers to propose laws that seem to govern biological networks (Gelfand, 2006). Different types of cellular networks (e.g. protein interaction and metabolic networks) seem to share with other complex bionetworks properties such as their “scale-free” nature and “small world” organization (Barabasi and Albert, 1999; Wagner and Fell, 2001; Koonin and Wolf, 2006).

In scale-free networks, a few nodes (hubs) have the largest number of connections to other nodes, whereas most of the nodes have just a few connections (Babu et al. 2006; Babu et al. 2007). This property is reflected in power-law distribution of connectivities. In practical terms, this relationship means that, in protein interaction networks, most proteins interact with a couple of others whereas a few proteins (hubs) interact with a large number of proteins, and that, in a metabolic network, a few molecules (hubs) participate in most reactions whereas the rest participate in one or two reactions. The “small world” concept refers to the property of such spoke-and-hub networks that the path length between nodes is short. This property means that a path of just a few interactions or reactions will connect almost any pair of molecules in the cell (Koonin, 2005; Koonin and Wolf, 2006). Understanding the additional levels in the hierarchy of biological networks and interactions between them will allow for integration of data and new theoretical predictions (Stearn and Magwene, 2003). Processes widely studied by evolutionary biologists such as selection, gene duplication, and neutral evolution are being examined in the context of network models as opposed to the level of individual genes or molecules, i.e. from data integration-based systems biology perspective. As researchers look more closely at the network structure, network features of higher order emerge. Networks with power-law distributions of connectivities can still differ in terms of clustering, motif frequency, nestedness, and fractal structure. However, the role that natural selection has in the evolution of network structures remains unknown (Babu and Aravind, 2006; Babu et al. 2007).

In recent years, bionetworks have been constructed using dynamic model based on time profiles of microarray data and ChIP-chip data via reverse engineering methods (Chen et al. 2004; Chang et al. 2005; Lin et al. 2005; Li et al. 2006; Wu et al. 2006; Chang et al. 2006; Chiang and Chao, 2007). Then, according to the dynamic models of biochemical networks and gene regulatory networks, the stability, robustness and noise filtering properties could be analyzed from the system theory perspective (Chen and Wang, 2006; Chen and Wu, 2006). Robustness is a ubiquitously observed property of biological systems (Freeman, 2000; Stelling et al. 2004; Chen et al. 2005). It is considered to be a fundamental feature of complex evolvable biological systems. It has been pointed out that robustness facilitates evolvability and robust traits are often selected by evolution (Kitano, 2004), i.e. complex biological systems must be robust against environmental and genetic perturbations to be evolvable. Evolution often selects bionetworks that might enhance the robustness of organisms (Kitano, 2004). The central role of biochemical networks in the cellular function provides a strong motivation to search the underlying principles of adaptive evolution in bionetworks. In order to test whether the physiological function would prevail under a new environment or not, the robustness and sensitivity criteria are developed to measure the tolerance of metabolite concentration variations of a biochemical network in face of environmental changes (Chen et al. 2007a). That is, these two robust criteria are derived as the selection forces for the metabolite networks to be preserved by natural selection in the evolutionary process. In fact, robustness allows changes in the structures and components of the biological system owing to gene mutations and environmental variations, but specific functions are maintained. Hence, robustness facilitates evolvability and evolution selects robust bionetworks (Yi et al. 2000; Kitano, 2004). By systems biology, the design principles of biochemical networks via natural selection in evolution are not in conceptual description but the underlying selection mechanisms should be investigated according to mathematical natural selection principles for biochemical networks (Chen et al. 2007a).

## Evolutionary Systems Biology: Integration Data-Based Approach

Network representations provide us with an excellent conceptual framework to understand the structure of transcriptional regulation, both at local and global level of organization. Several studies suggest that the graph theoretical principles of transcriptional networks could be employed to infer some of the significant properties of such networks for investigating the evolution of the transcriptional regulatory networks across diverse organisms (Janes and Yaffe, 2006).

Transcriptional regulators and their target genes form large gene regulatory networks. These and other molecular networks, such as protein interaction networks and metabolic networks, are intensely studied because their characterization has been greatly facilitated by new techniques in genomics and bioinformatics (Jungck et al. 2006). There has been significant progress in unraveling the transcriptional regulatory networks of various model organisms such as E. Coli and B. subtilis. By using computation methods such as orthology methods and binding-site profile methods, information of transcriptional regulatory networks is obtained from model organisms to poorly studied organisms by exploiting the publicly available completely sequenced genomes (Babu et al. 2006). The availability of the complete genome sequences of over 300 prokaryotes and the understanding of the structure of transcriptional regulatory networks have allowed us to address several fundamental questions on the evolution of transcriptional regulatory networks, which will provide us with an opportunity to identify the distinct evolutionary trends in shaping transcriptional regulatory networks at various levels of organization (Jungck et al. 2006; Babu et al. 2006).

The information regarding the structure of molecular networks opens a new dimension to the studies of molecular evolution because it allows inquiries that go beyond the evolution of individual genes. On one hand, we know that mutations at the level of individual genes influence the structure of these networks. On the other hand, natural selection acting on the global structure of networks may influence what kind of mutations can be tolerated on the gene level (Wagner, 2000, Sole et al. 2002). However, the structure of networks may influence the evolution of genes and vice versa. This interplay is part of the reason why network evolution is an intriguing and increasingly popular subject of evolution.

At present, we know very little empirically about the evolution of large genetic networks. More knowledge of the basic characterization of network structure and connectivity of genes is required for understanding the evolution of genes and the function of networks (Shiu and Borevitz, 2006). Regulatory motifs and their target genes which when put together reconstruct the entire network. Analysis of the conservation patterns of these network motifs across 175 genomes (Babu et al. 2006) revealed the contrary that network motifs are not conserved as complete units in other organisms. It was also found that organisms that were close evolutionary relatives did not conserve regulatory network motifs whereas several organisms that were distantly related conserved orthogolous network motifs. A comprehensive assessment for similarity in their network motif content revealed a statistically significant trend that organisms with similar lifestyle tend to regulate their target genes by means of similar network motifs (Barrett and Palsson, 2006). Several observations reveal an important principle in evolution of network motifs that orthologous genes in related organism living in different environments may require distinct patterns of gene expression by embedding them in an appropriate motif context in order to adapt better to the changing environment (Bjornstad and Harvill, 2005; Dekel and Alon, 2005; Fong et al. 2005; Babu and Aravind, 2006). That is, different organisms arrive at the best possible solutions to regulate the same gene by tinkering specific regulatory interactions (i.e. adjusting the edges among these nodes in the network) in order to optimize expression levels rather than by duplicating groups of genes that are already a part of a motif. In this context, other studies on duplicated genes within the transcriptional regulatory networks of E. Coli and yeast (Babu and Teichmann, 2003; Conant and Wagner, 2003; Teichmann and Babu, 2004) have shown that network motifs have not evolved by duplication of complete ancestral motifs, supporting the hypothesis that some interactions, which form a part of a motif in one organism, could have existed in different regulatory contexts in the ancestral genomes. Modular components make transcriptional networks very plastic during evolution and are able to readily incorporate new genes and new regulations. In fact, transcriptional networks can evolve rapidly: the edges in transcriptional networks appear to evolve on a faster time scale than the coding regions of genes. For example, related animals, such as mice and humans, have very similar genes, but the transcription regulation of these genes, which governs when and how much of each protein is made, is evidently quite different. In other words, many of the differences between animal species appear to lie in the differences in the edges of the transcriptional networks, rather than in the differences in their genes (Alon, 2007).

At the global level of evolutionary network structure, results from graph theoretical studies and the fact that global regulatory hubs control the expression of several genes suggest a trend that such hubs would assume importance in transcriptional networks and hence be more conserved in evolution than transcription factors. However, several analyses showed that there is no such trend and that regulatory hubs tend to evolve like any other transcription factors in the genome. This suggests that regulatory hubs have been independently innovated to regulate orthologous target genes in organisms living in different environments. These observations provide a strong support that hierarchical structure of these networks has converged to a similar scale-free topology, though with independent recruited regulatory hubs (Balaji et al. 2006; Pal et al. 2006; Korcsmaros et al. 2007). The reason may be that the binding affinity and specificity of a transcription factor and its target site can be affected by relatively small changes in the DNA-binding interface of the transcription factor, or in the binding site. As a result, DNA-binding domain could evolve new target sites relatively easily, resulting in rapid de novo emergence of new transcriptional interaction (Babu et al. 2006). Therefore, the extent of advantage conferred by orthologous transcription factors in terms of fitness to an organism might vary across organisms depending on the environment and hence during the course of evolution different proteins may emerge as global regulatory hubs in organisms. Then, through the emergence of different proteins as global regulatory hubs, transcriptional regulatory networks tend to approximate a scale-free topology. The overall scale-free topology is maintained, suggesting that such a structure has evolved convergingly and is an emergent property in evolution (Pal et al. 2006).

The above evolutionary network analyses are made by comparing topologies of transcriptional regulatory networks of different organisms, i.e. graphs with different nodes representing transcription factors and target genes, and directed edges connecting the former to the latter to represent the modification and diversification of the network structure of various organisms in the course of evolution. However, these approaches cannot gain insight into the dynamic mechanisms of networks in the evolutionary process, such as robust stability on mutations, sensitivity to environmental disturbance and noise filtering ability of gene networks, which are the main selection forces of the network evolution and play the important role of design principles of bionetworks in the evolutionary process from the systems biology perspective.

## Network Evolution: Dynamic Model Approach

Robustness is a ubiquitously observed property of biological systems. It is considered to be a fundamental feature of complex evolvable systems. It is pointed out that robustness facilitates evolvability and robust networks (or the corresponding traits) are often selected by evolution (Kitano, 2004), i.e. complex biological networks must be robust against environmental and genetic perturbations to be evolvable. Evolution often selects biological networks that might enhance robustness of the organism. In the past, most molecular biologists and biochemists assumed that variations in biological networks were due to historical accidents and natural selection. However, the design principles of biochemical networks via natural selection in evolution are still in conceptual description but not yet in mathematical rules. Can these mathematical natural selection principles for biological networks in evolution be unraveled? The investigation of design principles of biological networks in evolution is in its infancy and more underlying rules remain to be discovered. In fact, robustness allows changes in the structure and components of biological networks owing to intrinsic perturbations and extrinsic disturbances, but specific functions are still maintained (Chen et al. 2007a).

According to the hierarchical network interplaying of Figure 1, the higher-level selected networks will specify a selection force for lower levels. Just as external environment molds the evolution of local adaptation of organisms by natural selection, the internal biological network environment of populations is expected to head to the evolution of local biological networks in a favorable manner (Lynch and Walsh, 1998). Once the favored organisms are selected by natural selection, the lower-level networks have to maintain proper functions of the physiological systems of the favored organism. Therefore, the favored organisms are the selection force of the physiological systems. Since biochemical networks are the backbone of the physiological systems of organisms, in order to maintain the favored physiological system by natural selection, a biochemical network should be sufficiently robust to tolerate variations and environmental changes in the evolutionary process. Therefore, the favored physiological systems specify the selection force of biochemical networks. Similarly, in order to maintain the robustness of the favored biochemical networks, the gene regulatory network should be designed with enough robustness to tolerate gene mutations and to filter environmental noises. On the other hand, the lower-level networks will feedback to influence the higher-level networks in evolution. Therefore, the natural selection actually acts on the interplaying of the multiple networks. Hence, the revelation of robust adaptive design rules of biological networks by natural selection can gain much insight into the evolutionary mechanisms of biological networks (Chen et al. 2007a). The robust evolution principles of genetic networks and biochemical networks will be discussed in the following subsections.

### Adaptive design principles of genetic networks in the evolutionary process

As seen in Figure 1, the genetic network evolution lies at the lowest level of the biological network evolution. In order to maintain the proper function of the favored biochemical networks at high level, the lower-level genetic networks have to function properly. Therefore, robust stability is the natural selection force specified by the higher-level networks. Genetic networks are combined through feedback and feedforward loops so that normal favored cellular physiological and biochemical process in higher-level networks can be maintained in evolution. This intrinsic robustness of a genetic network enables co-option, so that new traits (or corresponding new genetic networks) can be generated in the evolutionary process (Kitano, 2004). The proposed robust adaptive rule via natural selection can mimic the evolution of genetic networks by computational simulation. For the convenience of illustration, we consider only the following linear genetic network (Chen and Wang, 2006)

1 $$\frac{dx(t)}{dt}\;=\;Nx(t)$$

where the concentration vector *x(t)* and stoichiometric matrix *N* are given, respecitvely by

$$x(t)\;=\;\left[\begin{array}{c} x_{1}(t)\\ \vdots \\ x_{n}(t) \end{array}\right],\;\;\;\;\;N\;=\;\left[\begin{array}{ccc} N_{11} & \cdots & N_{1n}\\ \vdots & \ddots & \vdots \\ N_{n1} & \cdots & N_{nn} \end{array}\right]$$

in which *x_i_* (*t*) denotes the concentration of the *i*th gene and *N_ij_* denotes the interaction between gene *j* and gene *i* in the genetic network.

Suppose the linear genetic regulatory network suffers from intrinsic molecular fluctuations due to thermal variation, alternative splicing, stochastic mutation, and so on (Graur and Li, 2000) so that stoichiometric matrix *N* is perturbed as *N* + Δ*N,* the intrinsic molecular fluctuation Δ*N* could be modeled as a Wiener process Δ*N = Mn*(*t*), where *M* denotes the locations and magnitudes of the perturbation and *n*(*t*) is a standard white Gaussian noise with unit variance to denote the stochastic part of the fluctuation (Zhang and Chen, 2006). That is, the stochastic part of the fluctuation is absorbed to *n*(*t*) with *dw*(*t*) = *n*(*t*)*dt. w*(*t*) is a standard Wiener process (or Brownian motion) to denote the stochastic process in the genetic fluctuation due to DNA mutations (McAdam and Arkin, 1997; McAdam and Arkin, 1999; Rao et al. 2002). Therefore, the genetic network under stochastic perturbation could be represented by (Chen and Wang, 2006)

2 dx(t) = Nx(t)dt + Mx(t)dw(t)

The last term *Mx*(*t*)*dw*(*t*) in (2) denotes the term due to intrinsic fluctuation Δ*Nx*(*t*). Because it is state-dependent, it will influence the stability of genetic network. In genetic network evolution, robust stability is the main selection force on genetic networks to tolerate this intrinsic fluctuation. According to stochastic Lyapunov stability (i.e. for a Lyapunov function *V*(*x*) > 0, $E\left(\frac{d}{dt}V(x)\right)\;\leq \;0$, where *E* denotes expectation. In the case of a linear system, we always choose) *V*(*x*) = *x^T^*(*t*)*Px*(*t*).), it has been shown that the perturbative genetic network in (2) is stable in probability if the following Lyapunov type equation (Chen and Wang, 2006)

3 $$PN\;+\;N^{T}P\;+\;M^{T}PM\;\leq \;0$$

has a symmetric positive definite solution *P* > 0.

### Remark 1

**Figure 2.**
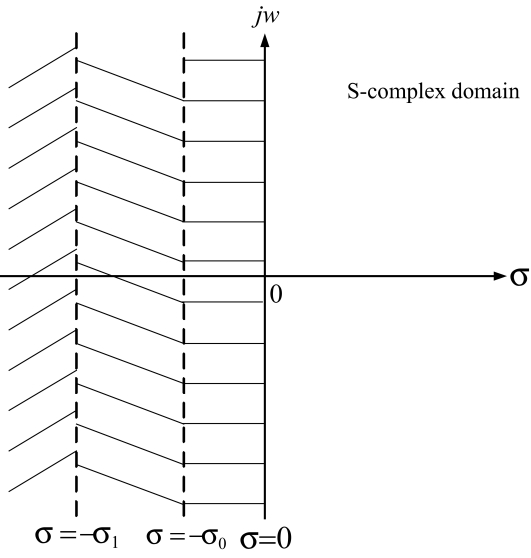
Stability region and robust stability region in *S* complex domain. All the eigenvalues on the left-hand side of *jw* axis (i.e. σ = 0) ensure that the network is stable. All the eigenvalues on the left-hand side of axis σ = −σ_0_ mean the network with stability margin (or robustness) σ_0_ If the eigenvalues are all on the left-hand side of axis σ = −σ_1_ it means the network is with robustness σ_1_ A genetic network with larger robustness means it can tolerate larger intrinsic genetic variations and filter larger extrinsic disturbances.

(i) For the intrinsic perturbation-free case, the stable condition is that the matrix inequality *PN* + *N^T^* *P* ≤ 0 has a symmetric positive solution *P >* 0. It means that all eigenvalues *λ_i_* = *σ_i_* + *jw_i_* of system interaction matrix *N* of a linear genetic network should be on the left-hand side of the s-complex domain (i.e. σ*_i_* ≤ 0 in Fig. 2) (Franklin et al. 1994; Qu, 1998). The existence of a symmetric positive solution *P >* 0 in (3) is stricter than that of *PN* + *N^T^* *P* ≤ 0 because the eigenvalues of the system matrix *N* should be located at the far left-hand side of the s-complex domain with large negative real values (i.e. σ*_i_* ≤ −σ_0_ in Figure 2, where the value of σ_0_ depends on the magnitude of perturbation *M*) in order to overcome the extra positive term *M^T^PM* due to intrinsic noises induced by genetic variations. If some eigenvalues of the system interaction matrix *N* of the genetic network are near the *jw* axis, then these modes (i.e. with eigenvalues in the zone between σ = 0 and σ = −σ_0_) are more easily perturbed by intrinsic molecular fluctuations across the *jw* axis such that the linear perturbed network becomes unstable and will be eliminated by natural selection in the evolutionary process. Therefore, the smallest distance from the locations of the eigenvalues of *N* to *jw* axis can be considered as the robustness measure of a linear genetic network (Franklin et al. 1994). For example, if the eigenvalues of *N* are all on the left-hand side of σ = −σ_0_ (see Fig. 2), then the robustness of this genetic network is σ_0_ If the magnitudes of intrinsic perturbations are larger than the robustness of the genetic network, it may be eliminated in the evolutionary process except when some new gene circuits are generated by intrinsic DNA mutations to improve its robustness.

If the genetic network also suffers from extrinsic disturbances *v*(*t*) outside the network, then the stochastic equation of the genetic network in (1) is modified as follows (Chen and Wang, 2006)

4 dx(t) = (Nx(t) + Hv(t))dt + Mx(t)dw(t)

where *H* is a coupling matrix which denotes the influence of the environmental disturbance on the state *x*(*t*) of the genetic network.

In this situation, the natural selection acting on the genetic network lies in the tolerance of the intrinsic noises due to mutations and the filtering ability of extrinsic disturbances *v*(*t*) to maintain the robust properties of the genetic network to guarantee the proper functions of high-level biochemical networks and physiological systems. In this case, the robust filtering of the genetic network is defined as follows (Zhang et al. 2005)

5 $$\frac{∥x(t){∥}_{2}}{∥v(t){∥}_{2}}\;\leq \;\rho \;\text{or}\;∥x(t){∥}_{2}\;\leq \;\rho \;∥v(t){∥}_{2}$$

for some positive *ρ*. If *ρ* < 1 we say that the extrinsic disturbance is filtered (attenuated) by the genetic network. In order to tolerate the intrinsic fluctuation and to avoid the influence of environmental disturbances *v*(*t*) to achieve a filtering ability *ρ*< 1 in (5), the genetic network should satisfy the following inequality (Chen and Wang, 2006)

6 $$PN\;+\;N^{T}P\;+\;M^{T}PM\;+\;I\;+\;\frac{1}{\rho^{2}}\;PHH^{T}\;P\;\leq \;0$$

In order to achieve a robust filtering ability to tolerate the intrinsic noise and to attenuate the extrinsic disturbance to maintain the function of a genetic network, all the eigenvalues of system interaction network *N* of a gene network should be further on the left-hand side of the s-complex domain, for example on the left-hand side with σ ≤−σ_1_ in Figure 2 (Franklin et al. 1994). That is, the genetic networks with eigenvalues in the zone between σ = 0 and σ = σ_1_ may be eliminated by natural selection under the requirement of robust stability and filtering ability *ρ* < 1 Therefore, the robust stability and filtering ability according to the inequality in (6) could be considered as a rule or mechanism of natural selection for linear genetic networks in evolution. If a genetic network cannot satisfy the robustness and filtering requirement of (6), then it cannot tolerate intrinsic fluctuations due to mutations or filter the external disturbances and will thus be eliminated by natural selection. The above evolution analysis is performed only on the linear genetic network. However, in practical genetic networks, their dynamic equations are always nonlinear. In order to consider the nonlinear stochastic regulatory networks, the linear stochastic system in (4) should be generalized as the following Langevin equation (Qu, 1998; Rao et al. 2002)

7 dx(t) = (N(x) + H(x)v(t))dt + M(x)dw(t)

where *N*(*x*) denotes the nonlinear interactions of nonlinear genetic regulatory networks, *M*(*x*)*dw*(*t*) denotes the nonlinear intrinsic fluctuations due to genetic mutations and *H*(*x*)*v*(*t*) denotes the external disturbances *v*(*t*) through a coupling matrix *H*(*x*). Recent studies have found that the dynamic model of genetic network in (7) could be identified by microarray data via the so-called reverse engineering methods (Tsai et al. 2005; Ko et al. 2006).

In general, the phenotype of a nonlinear genetic network is close to one of the equilibrium points of Langevin equation in (7). If a specific equilibrium point of phenotype is of interest, the origin should be shifted to that equilibrium point for the convenience of discussion. In evolution, the genetic network in (7) must have enough robust stability and filtering ability to tolerate both intrinsic noises and extrinsic disturbances to maintain their functions, or they will be shifted to another equilibrium point to change their phenotype or be eliminated by natural selection.

If the nonlinear genetic network of (7) satisfies the robust filtering requirement in (5), it has robust stability to tolerate intrinsic noises and filtering ability to attenuate external disturbances to a desired level *ρ*. From the results in (Chen and Wang, 2006), according to nonlinear robust filtering theory (Zhang et al. 2005), it is found that if the following inequality holds for some *V*(*x*) > 0,

8 $$\begin{array}{c} x^{T}x\;+\;{\left(\frac{\partial V(x)}{\partial x}\right)}^{T}\;N(x)\;+\;\frac{1}{2}\;M^{T}(x)\frac{\partial^{2}V(X)}{\partial^{2}X}M(x)\\ +\;\frac{1}{4\rho^{2}}{\left(\frac{\partial V(x)}{\partial x}\right)}^{T}\;H(x)H^{T}(x)\frac{\partial V(x)}{\partial x}\;\leq \;0 \end{array}$$

then the robust stability and filtering ability in (5) is achieved in evolution, i.e. the inequality in (8) is the mechanism of natural selection for nonlinear genetic networks. If it is violated, the phenotype of the nonlinear genetic network will be shifted from this equilibrium point to another equilibrium point or it will be eliminated by natural selection.

In general, it is not easy to solve the nonlinear inequality of natural selection in (8) to gain more insight into the adaptive evolution mechanism of nonlinear genetic networks. In this situation, for a better understanding, the global linearization technique is employed to discuss the selection force in (8) of the nonlinear genetic network in (7).

**Figure 3.**
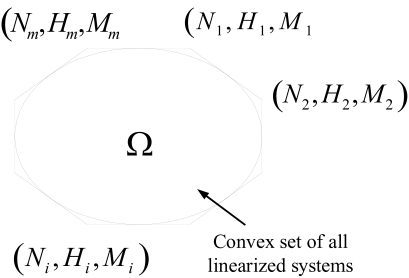
The generalized linear systems are all in the convex set Ω and (*N_i_, H_i_, M_i_*), *i* = 1, …, *m* denoted the system parameters of the vertices of the convex hull *C*_0_{…} of Ω. Then the robust stability and filtering ability of the nonlinear system could be characterized by these linear systems in the vertices.

Suppose the global linearization of *N*(*x*), *H*(*x*) and *M* (*x*) in (7) is defined as

$$\frac{\partial }{\partial x}\left[\begin{array}{c} N(x)\\ H(x)\\ M(x) \end{array}\right]\;\subset \;\Omega \;\in \;R^{3n\times n}$$

for all *x*(*t*) and suppose the polytope Ω of all linearized systems is bounded by the following convex hull with *m* vertices (see Fig. 3)

$$\Omega \;\in \;C_{0}\;\left\{\left[\begin{array}{c} N_{1}\\ H_{1}\\ M_{1} \end{array}\right]\;,\;\cdots \;,\;\left[\begin{array}{c} N_{m}\\ H_{m}\\ M_{m} \end{array}\right]\right\}$$

where *C*_0_ { } denotes the convex hull consisted of vertices {…}. Therefore, the nonlinear genetic network in (7) can be interpolated and represented by the following *m* linearized genetic networks at vertices (Boyd et al. 1994)

10 $$\begin{array}{l} dx(t)\;=\;\left(N_{i}x(t)\;+\;H_{i}v(t)\right)dt\\ \;\;\;\;\;\;\;\;\;\;\;\;\;\;\;+\;M_{i}x(t)dw(t),\;\;\;\;\;\;\;\;i\;=\;1,\;\cdots \;,m \end{array}$$

Then, according to the linear robust stability and filtering ability (Chen and Wang, 2006), if the following inequalities are all satisfied for a common *ρ* > 0

11 $$\begin{array}{l} PN_{i}\;+\;N_{i}^{T}P\;+\;M_{i}^{T}PM_{i}\;+\;I\\ \;\;\;\;\;\;\;\;\;\;+\;\frac{1}{\rho^{2}}PH_{i}H_{i}^{T}P\;\leq \;0,\;\;\;\;\;\;\;i\;=\;1,\;\cdots \;,m \end{array}$$

then both the robust stability and filtering ability *ρ* are achieved for the nonlinear genetic network (7) in evolution, i.e. the robust stability and filtering ability according to the *m* inequalities in (11) are the natural selection rules in evolution, which could be considered as an equivalent linear solution to the nonlinear inequality in (8). In the natural selection rule in (11), all the eigenvalues of *N_i_* of all linearized genetic networks at the vertices in (10) should be all on the left-hand side of the axis σ = −σ_1_ in Figure 2 so that the nonlinear genetic network in (7) has enough robustness to tolerate intrinsic variations due to mutations and to filter environmental disturbances in evolution to avoid extinction by natural selection. The genetic mutations, which could lead to feedback gene circuits to improve robustness and filtering ability, i.e. to satisfy the selection force in (11) are favored by natural selection in the evolutionary process. However, some mutations, which could make the corresponding internal variation matrices *M_i_* and coupling matrices *H_i_* small, such as redundancy, duplication and self-regulation so that the selection rule in (11), is not easy to violate due to these buffers, are also favored by natural selection (Isaacs et al. 2003; Langkjaer et al. 2003; Kellis et al. 2004; Teichmann and Babu, 2004).

If *ρ* < 1in (11), the eigenvalues of *N_i_* should be in the far left-hand side (i.e. σ_1_ is large) to attenuate the environmental noise. If *ρ* > 1, it is easier for *N_i_* to satisfy the filtering ability in (11). In this situation, the genetic network is much influenced by the environmental noise to easily move toward another equilibrium point so that the genetic network is more adaptive to the environmental changes in the evolutionary process, especially for *ρ* ≫ 1, to generate a new phenotype to a new environment. This is a tradeoff between robust stability and adaptability of genetic networks in the evolutionary process.

### Adaptive design principles of the biochemical networks in evolutionary process

The central role of the biochemical networks in cellular function provides a strong motivation to search for the underlying principles of the adaptive evolution of biochemical networks. As seen in Figure 1, the favored physiological systems will lead to a selection force on biochemical networks in the evolutionary process. Because the biochemical networks are the backbone of the physiological systems, they should have enough robustness to tolerate parameter variations and less sensitivity to attenuate the influence of external disturbance so that the favored physiological functions could be preserved in the evolutionary process. In order to test whether a physiological function would prevail under a new environment or not, the robustness and sensitivity criteria have been developed to measure the tolerance of the variations in metabolite concentration of a biochemical network in face of environmental changes (Chen et al. 2005). The design mechanisms for the biochemical networks by selection force in the evolutionary process will be described as follows.

The dynamic system of a biochemical network can be represented in the following S-system (Savageau, 1976; Voit, 2000).

12 $${\dot{x}}_{i}\;=\;\alpha_{i}\prod_{j=1}^{n+m}x_{j}^{g_{ij}}\;-\;\beta_{i}\prod_{j=1}^{n+m}x_{j}^{h_{ij}},\;\;\;\;\;i\;=\;1,\;\ldots ,\;n$$

where *x*_1_,…, *x_n_*_+_*_m_* are metabolites, such as substrates, enzymes, factors or products of a biochemical network, in which *x*_1_,…, *x_n_* denote the *n*-dependent variables (intermediate metabolites and products) and *x_n_*_+1_,…, *x_n_*_+_*_m_* denote the independent variables (initial reactants and enzymes), α*_i_* and β*_i_* denote the rate constants, and *g_ij_* and *h_ij_* represent the kinetic parameters of the biochemical network. These parameters could be estimated by experimental data or microarray data (Tsai and Wang, 2005; Ko et al. 2006).

The evolutionary time is much longer than the transient time of the nonlinear biochemical network in (12) and the phenotype of a biochemical network is close to the steady state. Therefore, for simplicity, we shall focus on the relationship between the robustness and evolution design principles of a biochemical network at the steady-state case.

Consider the steady state of biochemical network in (12), we get

13 $$\alpha_{i}\prod_{j=1}^{n+m}x_{j}^{g_{ij}}\;=\;\beta_{i}\prod_{j=1}^{n+m}x_{j}^{h_{ij}},\;\;\;i\;=\;1,\;2,\;\ldots \;,\;n$$

Taking the logarithm on both sides of (13), introducing new variable $y_{j}\;=\;\text{ln}\;x_{j},\;a_{ij}\;=\;g_{ij}\;-\;h_{ij},\;b_{i}\;=\;\text{ln}\;\left(\frac{\beta_{i}}{\alpha_{i}}\right)$, and after some rearrangements, we get

14 $$\begin{array}{ccc} a_{11}y_{1}\;+\;a_{12}y_{2}\;+\;\cdots \cdots \;+\;a_{1n}y_{n} & = & b_{1}\;-\;a_{1,n+1}y_{n+1}\;-\;\cdots \cdots \;-\;a_{1,n+m}y_{n+m}\\ a_{21}y_{1}\;+\;a_{22}y_{2}\;+\;\cdots \cdots \;+\;a_{2n}y_{n} & = & b_{2}\;-\;a_{2,n+1}y_{n+1}\;-\;\cdots \cdots \;-\;a_{2,n+m}y_{n+m}\\ a_{31}y_{1}\;+\;a_{32}y_{2}\;+\;\cdots \cdots \;+\;a_{3n}y_{n} & = & b_{3}\;-\;a_{3,n+1}y_{n+1}\;-\;\cdots \cdots \;-\;a_{3,n+m}y_{n+m}\\ \vdots & \vdots & \vdots \\ a_{n1}y_{1}\;+\;a_{n2}y_{2}\;+\;\cdots \cdots \;+\;a_{nn}y_{n} & = & b_{n}\;-\;a_{n,n+1}y_{n+1}\;-\;\cdots \cdots \;-\;a_{n,n+m}y_{n+m} \end{array}$$

The above equations could be represented by the following steady-state equation (Voit, 2000)

15 $$A_{D}Y_{D}\;=\;b\;-\;A_{I}Y_{I}$$

where

$$\begin{array}{l} Y_{D}\;=\;\left[\begin{array}{c} y_{1}\\ \vdots \\ y_{n} \end{array}\right],\;b\;=\;\left[\begin{array}{c} b_{1}\\ \vdots \\ b_{n} \end{array}\right],\;Y_{I}\;=\;\left[\begin{array}{c} y_{n+1}\\ \vdots \\ y_{n+m} \end{array}\right],\\ A_{D}\;=\;\left[\begin{array}{ccc} a_{11} & \cdots & a_{1n}\\ \vdots & \ddots & \vdots \\ a_{n1} & \cdots & a_{nn} \end{array}\right],\;A_{I}\;=\;\left[\begin{array}{ccc} a_{1,n+1} & \cdots & a_{1,n+m}\\ \vdots & \ddots & \vdots \\ a_{n,n+1} & \cdots & a_{n,n+m} \end{array}\right] \end{array}$$

in which *A_D_* denotes the system matrix of the catalytic interactions among the dependent variables *Y_D_* and *A_I_* indicates the catalytic interactions between the dependent variables *Y_D_* and the independent variables *Y_I_* (i.e. the environmental medium of the metabolic system). From the simple algebraic steady-state equation in (15), obviously, the S-system in (12) is a useful model for describing the phenotype of biochemical networks (Voit, 2000).

If the inverse of *A_D_* exists, the steady state (or phenotype) of the biochemical network is solved by (Voit, 2000)

16 $$Y_{D}\;=\;A_{D}^{-1}(b\;-\;A_{I}Y_{I})$$

The steady state *Y_D_* in (16) is one of the equilibrium points of the nonlinear biochemical network in (12). Actually, there are many equilibrium points for (12), which represent different phenotypes. Only the equilibrium point (or phenotype) in (16) is favored by natural selection in the evolution via specification of parameters *A_D_, b, A_I_* and environmental enzymes *Y_I_* in (16). Biochemical networks perform their physiological functions within some local regions of the equilibrium point. In the evolutionary process, suppose that there exist some parameter variations, Δα*_i_*, Δβ*_i_*, Δ*h_ij_*, Δ*g_ij_*, and Δ*Y_I_* in (12), which could be considered as design parameters in the evolutionary process owing to genetic mutations or environmental changes (Graur and Li, 2000). Then the corresponding perturbative steady state of the biochemical network is given by (Chen et al. 2005)

17 $$\begin{array}{l} \left(A_{D}\;+\;\Delta A_{D})(Y_{D}\;+\;\Delta Y_{D}\right)\\ \;\;\;\;=\;(b\;+\;\Delta b)\;-\;(A_{I}\;+\;\Delta A_{I})(Y_{I}\;+\;\Delta Y_{I}) \end{array}$$

Because the biochemical networks are the backbone of physiological systems of organisms, a biochemical network should be sufficiently robust to tolerate the variations due to genetic mutations and environmental changes to maintain its function properly in the evolutionary process. It is found that if the following robustness condition holds (Chen et al. 2005)

18 $$∥A_{D}^{-1}\Delta A_{D}{∥}_{2}\;<\;1\;\;\;or\;\;\;\Delta A_{D}\Delta A_{D}^{T}\;<\;A_{D}A_{D}^{T}$$

then the phenotype of the perturbed genetic network exists as follows

19 $$\begin{array}{l} Y_{D}\;+\;\Delta Y_{D}\;=\;{\left(A_{D}\;+\;\Delta A_{D}\right)}^{-1}[(b\;+\;\Delta b)\\ \;\;\;\;\;\;\;\;\;\;\;\;\;\;\;\;\;\;\;\;\;\;\;-(A_{I}\;+\;\Delta A_{I})(Y_{I}\;+\;\Delta Y_{I})] \end{array}$$

If the robustness condition is violated, the steady state of the perturbed biochemical network in (19) may cease to exist or move to another equilibrium point with a change of the phenotype. The changes Δ*b*, Δ*Y_I_*, and Δ *A_I_* will influence the phenotype variation Δ*Y_D_* in (19). Their effects on the phenotype have been discussed by the following sensitivity analysis of biochemical network (Voit, 2000; Chen et al. 2005).

20 $$\frac{\Delta Y_{D}}{\Delta b}\;=\;A_{D}^{-1},\;\;\;\frac{\Delta Y_{D}}{\Delta Y_{I}}\;=\;-A_{D}^{-1}A_{I},\;\;\;\frac{\Delta Y_{D}}{\Delta A_{I}}\;=\;-A_{D}^{-1}Y_{I}$$

In order to tolerate the variations Δ*b*, Δ*Y_I_*, and Δ*A_I_* to preserve the favored phenotype of a biochemical network in the evolutionary process, the sensitivities in (20) should be below some values as follows (Chen et al. 2007a):

21 $${\left\|\frac{\Delta Y_{D}}{\Delta b}\right\|}_{2}\;\leq \;s_{1},\;\;\;{\left\|\frac{\Delta Y_{D}}{\Delta Y_{I}}\right\|}_{2}\;\leq \;s_{2},\;\;\;{\left\|\frac{\Delta Y_{D}}{\Delta A_{I}}\right\|}_{2}\;\leq \;s_{3}$$

or equivalently

22 $$I\;\leq \;s_{1}^{2}A_{D}A_{D}^{T},\;\;\;A_{I}A_{I}^{T}\;\leq \;s_{2}^{2}A_{D}A_{D}^{T},\;\;\;Y_{I}Y_{I}^{T}\;\leq \;s_{3}^{2}A_{D}A_{D}^{T}$$

where *s*_1_, *s*_2_ and *s*_3_ are some small sensitivity values so that the phenotypes *Y_D_* *+* Δ*Y_D_* of perturbed biochemical networks would not change too much in (19) in comparison with the nominal values in (16) (i.e. Δ*Y_D_* in (19) should be small enough) and can be favored by natural selection.

### Remark

- According to the above analyses, the perturbed biochemical networks with parameter variations that violate the robustness criterion in (18) will be eliminated by natural selection. Therefore, the perturbed biochemical network should satisfy the robustness criterion in order to guarantee not to be perturbed too much from its equilibrium (for the normal physiological function) in the evolutionary process. Because the violation of (18) means a lethal perturbation, the robustness criterion in (18) is the necessary condition for survival under natural selection (Chen et al. 2007a). From the robustness criterion in (18), natural selection favors the perturbed biochemical networks with small perturbations $\Delta A_{D}\Delta A_{D}^{T}$ so that the robustness criterion is not violated. A biochemical network with redundancy and self-regulation can attenuate perturbation Δ *A_D_*. Further, a biochemical network with adequate negative feedbacks can increase $A_{D}A_{D}^{T}$ to tolerate large parameter variations $\Delta A_{D}\Delta A_{D}^{T}$ in the evolutionary process. These robust adaptive designs with feedbacks are also favored by natural selection in the evolutionary process of biochemical networks. This is why there is so much redundancy due to duplicated genes, modularity, self-regulation and feedback pathways in the biochemical networks in organisms (Isaacs et al. 2003; Langkjaer et al. 2003; Kellis et al. 2004; Teichmann and Babu, 2004). A scale free structure could reduce the effect of $\Delta A_{D}\Delta A_{D}^{T}$ on the stability of bionetworks and is favored by nature selection in evolution.
- The sensitivity criteria in (21) or (22) determine the ranges of the sensitivities of phenotype change Δ*Y_D_* to parameter variations and environmental changes by natural selection in the evolutionary process. For a functional biochemical network, it should satisfy the sensitivity criteria to prevent the metabolite concentration from being changed too much by environmental changes. Hence, the steady state (phenotype) of a biochemical network can be preserved while exposing the parameter variation and environmental changes to natural selection in evolutionary process. The assumption that the three sensitivity criteria in (21) all hold for natural selection is derived from the fact that biochemical networks are the backbone of physiological systems and cannot be too sensitive to environmental changes especially for some core (conserved) biochemical networks. Actually, the specification of sensitivities *s_i_*, *i* = 1, 2, 3 in (21) or (22) is case by case. If some sensitivity criteria in (21) are relaxed, i.e. some of the inequalities in (21) are violated or some *s_i_* are specified much larger, the phenotypes with changes to some environmental variations will also be favored by natural selection. In this situation, the phenotype of biochemical networks are much influenced by environmental variations to move toward another equilibrium so that they may be more adaptive to environmental changes in the evolutionary process. In this case, new phenotypes are more easily generated in order to be more adaptive to the new environment. This is a tradeoff between robustness and adaptability in biochemical networks in the evolutionary process.
- The robustness criterion in (18) and sensitivity criteria in (21) are called the adaptive design rules of natural selection on biochemical networks in the evolutionary process. There are many perturbed biochemical networks that can satisfy the adaptive design rules of natural selection in the evolutionary process (Chen et al. 2007a). If they are selected by natural selection, there are some differences in phenotype among these selected biochemical networks. After several generations in the evolutionary process, due to co-option of existing biochemical networks, diversities of the biochemical networks with conserved physiological function but with different structures will be developed (Freeman, 2000). This is the origin of the diversities of biochemical networks within organisms in evolution. However, if the requirements on the robustness in (18) and sensitivities in (21) are stricter (or more conservative), only a few solutions (or structures) can be selected by natural selection to meet these requirements. This is the reason why a conserved core biochemical network has less diversity (Chen et al. 2007a).

## Network Evolution from Synthetic Biology Point of View

The adaptive design rules of natural selection in the evolutionary process not only gain much insight into the evolutionary mechanism but also provide systematic robust genetic and biochemical circuit design methods of synthetic biology for biotechnological and therapeutic purpose in the future. Since systems biology is a foundation for genomescale synthetic biology, it will provide insight into the evolution of cellular systems. These insights will illustrate how changes in network topology, biochemical parameter values due to biochemical alterations, and molecular structures mediating the evolution of circuits. High-throughput measurements will be necessary for determining what is evolved and how engineering genetic networks *in vivo* are contributed by mimicking the evolutionary process of genetic networks (Hasty et al. 2000; Hasty et al. 2002; Savageau, 2001; Voit, 2003). An approach involving directed evolution has been applied to rationally designed synthetic network *in vivo*. In this case, the synthetic circuit was already well described and the target mutation, together with screening according to fluorescent reporter properties, was employed to make a nonfunctional circuit functional. This synthetic biology involved a combination of the rational circuit design and evolution to improve performance (Altamirano et al. 2000; Wang et al. 2000; Farmer and Liao, 2000). Unfortunately, synthetic biologists will always be confronted by the inaccuracies of their design algorithms in modeling reality, with its vast number of biochemical parameters (Benner and Sismour, 2005; Church, 2005; Sprinzak and Elowitz, 2005). To over this drawback, the adaptive design algorithm in the above section could be modified to guarantee a desired robust stability and filtering ability of synthetic biological networks.

### Application of evolutionary systems biology to robust genetic network design

Consider the stochastic perturbative genetic network in (2). If the perturbative genetic network does not have enough robust stability to tolerate stochastic parameter variations due to genetic mutation, then some feedback circuits *F* are designed to robustly stabilize the perturbative genetic network as follows (Chen and Wu, 2006)

23 dx(t) = (N + F) x(t)dt + Mx(t)dw(t)

where

$$F\;=\;\left[\begin{array}{ccc} F_{11} & \cdots & F_{1n}\\ \vdots & \ddots & \vdots \\ F_{n1} & \cdots & F_{nn} \end{array}\right].$$

*F_ij_* denotes the designed feedback interaction from gene *j* to gene *i*, which could be implemented through transformation and transfection biotechnologies. *F_ij_* = 0 if no feedback design exists from gene *j* to gene *i*.

According to stochastic Lyapunov stability as (3), if the following inequality holds, for *P = P^T^* > 0 (Chen and Wu, 2006)

24 $$P(N\;+\;F)\;+\;{\left(N\;+\;F\right)}^{T}\;\;P\;+\;M^{T}\;PM\;\leq \;0$$

then the perturbative genetic network is robustly stabilized by the feedback circuit design *F.*

The feedback circuit design *F* in (24) could be considered as the circuits favored by the designer rather than by natural selection in evolution. Therefore, it is a potential application of network evolution to synthetic biology. In synthetic biology, the feedback design *F* satisfying the requirement in (24) can guarantee the genetic circuit to tolerate intrinsic stochastic parameter perturbations *Mx*(*t*)*dw*(*t*) in (23).

If the perturbative genetic network in (23) also suffers from external disturbances as follows (Chen and Wu, 2006)

25 dx(t) = (N + F) x(t)dt + Mx(t)dw(t) + Hv(t)dt

and the filtering ability *ρ* cannot be achieved, then the feedback circuit *F* to be designed not only should tolerate intrinsic parameter variations but also filter environmental disturbances. According to the robust filtering in (6), if *F* is specified such that the following inequality holds for *P = P^T^* > 0

26 $$\begin{array}{l} P\left(N\;+\;F\right)\;+\;{\left(N\;+\;F\right)}^{T}\;P\;+\;M^{T}PM\\ \;\;\;\;\;+\;I\;+\;\frac{1}{\rho^{2}}\;PHH^{T}\;P\;\leq \;0 \end{array}$$

then the robust filtering ability *ρ* in (5) is guaranteed, i.e. the feedback circuit *F* should be designed in (25) to satisfy the inequality (26) (Chen and Wu, 2006).

If the nonlinear genetic network in (7) cannot achieve robust stability and filtering ability *ρ*, the feedback circuit *F* should be designed to improve its robust stability and filtering ability

27 dx(t) = (N(F, x) + H(x)v(t))dt + M(x)dw(t)

where *N*(*F*, *x*) denotes merges of feedback circuit into nonlinear *N*(*x*). By a similar method as in (10) and (11), if the following inequalities hold for common *P = P^T^* > 0

28 $$\begin{array}{l} PN_{i}(F)\;+\;N_{i}^{T}(F)P\;+\;M_{i}^{T}\;PM_{i}\;+\;I\\ \;\;\;\;\;+\;\frac{1}{\rho^{2}}\;PH_{i}H_{i}^{T}\;P\;\leq \;0,\;\;\;\;\;i\;=\;1,\;\cdots \;,m. \end{array}$$

then the robust stability and filtering ability in (5) is guaranteed, where *N_i_* (*F*) denotes the matrix *N_i_* that contains the elements of matrix *F*. Therefore, the robust circuit design principle is to select adequate feedback circuits *F* so that the inequalities in (28) have a common solution *P* > 0 The above is an application of evolutionary systems biology to the robust genetic network design from the synthetic biology perspective, with the designer, instead of natural selection, selecting *F* in the design procedure. In the next subsection, application of evolutionary systems biology to robust biochemical networks will be introduced from the synthetic biology perspective.

### Application of evolutionary systems biology to robust biochemical network design

The robustness criterion in (18) and sensitivity criteria in (21) may be violated by large parameter perturbations δ*A_D_*, δ*b*, and enzyme change δ*Y*_1_ due to genetic mutations, environmental changes and diseases in the evolutionary process. According to the design principles of natural selection, a robust design method in the above section can be developed for biochemical networks to improve robustness to compensate parameter perturbations and to attenuate external disturbances, which are useful for synthetic biologists when the synthetic biochemical networks have to work robustly with proper functions under both intrinsic variations and extrinsic disturbances.

Suppose a robust circuit control design is developed for a biochemical network in (12) by a state feedback method as follows (Chen et al. 2007b)

29 $${\dot{x}}_{i}\;=\;\alpha_{i}\prod_{j=1}^{n+m}x_{j}^{g_{ij}}\prod_{k=1}^{n}x_{k}^{f_{ik}}\;-\;\beta_{i}\prod_{j=1}^{n+m}x_{j}^{h_{ij}}\prod_{k=1}^{n}x_{k}^{e_{ik}}\;\;i\;=\;1,\;\ldots \;,n$$

where $x_{k}^{f_{ik}}$ denotes a biochemical control circuit via *x_k_* for regulating the production of *x_i_* with a kinetic parameter *f_ik_* and $x_{k}^{e_{ik}}$ denotes a biochemical control circuit via *x_k_* for regulating the degradation of *x_i_* with the kinetic parameter *e_ik_* .

The choice between regulating objects, and *x_k_* and *x_i_* the specification of kinetic parameters, *f_ik_* and *e_ik_*, are to be made according to the feasibility of biochemical circuit linkages to achieve both the robust stability to tolerate Δ*A_D_* within a prescribed range of kinetic parameter perturbations and the desired sensitivities to attenuate the other parameter perturbations Δ*b*, Δ*A_I_*, and the environmental disturbance Δ*Y*_l_ Since *f_ik_* and *e_ik_* are the elasticities of the corresponding enzymes in the designed control circuits, the implementation of control circuits are highly dependent on the specification of elasticities of these enzymes (Chen et al. 2007b).

According to the robustness analysis in the above section, a biochemical circuit design scheme for robust control of biochemical networks is developed. Consider the robust control systems of the biochemical network in (29). Using a similar procedure from (13) to (17), we obtain

30 $$\begin{array}{l} \left(A_{D}\;+\;F\;+\;\Delta A_{D})(Y_{D}\;+\;\Delta Y_{D}\right)\\ \;\;\;\;\;=\;(b\;+\;\Delta b)\;-\;(A_{I}\;+\;\Delta A_{I})(Y_{I}\;+\;\Delta Y_{I}) \end{array}$$

where the control parameter matrix is defined as

31 $$F\;=\;\left[\begin{array}{ccc} f_{11}\;-\;e_{11} & \cdots & f_{1n}\;-\;e_{1n}\\ \vdots & \ddots & \vdots \\ f_{n1}\;-\;e_{n1} & \cdots & f_{nn}\;-\;e_{nn} \end{array}\right]$$

where *f_ik_* and *e_ik_* are the kinetic parameters of the biochemical control circuits to be specified in (29).

By the robustness criterion in (18), if

32 $$∥{\left(A_{D}\;+\;F\right)}^{-1}\;\Delta A_{D}{∥}_{2}\;<\;1\;or\;\Delta A_{D}\Delta A_{D}^{T}\;<\;\left(A_{D}\;+\;F\right)\;{\left(A_{D}\;+\;F\right)}^{T}$$

then the phenotype of the perturbed biochemical network is preserved, i.e. the circuit control *F* can improve the robustness to tolerate larger parameter perturbations Δ*A_D_* for a biochemical network. Similarly, if the desired sensitivity of biochemical networks cannot be achieved to attenuate variation of the other parameters and external disturbances in (21), the circuit control *F*could be designed as follows (Chen et al. 2007b)

33 $$\begin{array}{l} I\;\leq \;s_{1}^{2}\;\left(A_{D}\;+\;F\right)\;{\left(A_{D}\;+\;F\right)}^{T},\\ A_{I}A_{I}^{T}\;\leq \;s_{2}^{2}\;\left(A_{D}\;+\;F\right)\;{\left(A_{D}\;+\;F\right)}^{T},\\ Y_{I}Y_{I}^{T}\;\leq \;s_{3}^{2}\;\left(A_{D}\;+\;F\right)\;{\left(A_{D}\;+\;F\right)}^{T} \end{array}$$

to achieve some desired sensitivities *s*_1_, *s*_2_ and *s*_3_ in (21) so that the phenotypes of the perturbed biochemical network will not change too much.

According to the above analyses made using the design principles of natural selection in evolution, if we specify circuit control matrix *F* of the biochemical network in (29) to satisfy the requirements of robustness in (32) and sensitivities in (33), then the biochemical network could tolerate prescribed ranges of parameter variations and also achieve desired sensitivities. These are potential applications of network evolution to robust design of synthetic biochemical networks in the future.

## Discussion and Conclusion

The ultimate goal of systems biology is to achieve an integrated understanding of life forms with all their complex characteristics at multiple levels. An important step of systems biology is to create biologically realistic models of network formation, evolution and function. From a more general perspective, evolutionary systems biology focuses on one core problem of systems biology, the evolutionary interplay between the genotype and phenotype. It is interesting that selected units in biological networks are not genetic sequences, but topological properties of the networks. The interplay between the selection forces and selected robust properties in multi-level biological network is a significant point of systems biology. Network evolution encompasses many different computational approaches and dynamical models to deal with the analyses of vast experimental data, especially microarray data, for probing and understanding the evolution of biological systems. These computational approaches and models have multiple applications in synthetic biology, with potential for genetic circuit designs and drug designs (Chen and Li, 2007).

For evolutionary biological networks, the complex interplaying of biological systems poses a tremendous challenge to evolutionary systems biologists, as does the uncertain and noisy nature of large-scale data. The first challenge that evolutionary systems biologists must overcome is the construction of biological system models according to genetic networks and protein-protein interaction networks by means of microarray data. With respect to the structures of genetic networks and protein-protein interaction networks, the second challenge arises from how to get the information of gain and loss of network components by mutation and natural selection in the evolutionary process of bio-networks.

Concerning analyses of system dynamics and selection forces of an evolutionary biological network, another challenge arises from the fact that many biological systems are nonlinear. For nonlinear stochastic genetic networks or biochemical networks, some advanced nonlinear robust stability theory and filtering theory is necessary for the analyses of evolutionary systems biology and its application to synthetic biology. Obviously, the proposed global linearization method are useful to analyze the robustness and filtering of the nonlinear biological networks in the evolutionary process. Recently, fuzzy approximation theory becomes also an efficient method to treat nonlinear robust stabilization and filtering ability of nonlinear stochastic systems (Chen et al. 2007c). It may be a potential method for network evolution and synthetic biology.
